# Supplementary material for: Efficient and Stable Deep-Blue 0D Copper-Based Halide TEA2Cu2I4 with Near-Unity Photoluminescence Quantum Yield for Light-Emitting Diodes
Source: Nanomaterials (Basel). 2024 Nov 28;14(23):1919. doi: 10.3390/nano14231919 (PMC11643752; doi:10.3390/nano14231919)
Supplement: Supplementary file 1 [file nanomaterials-14-01919-s001.zip › nanomaterials-3283310-supplementary.pdf]

nanomaterials

Supporting Information

Efficient and Stable Deep-Blue 0D Copper-Based Halide $TEA_2Cu_2I_4$ with Near-Unity Photoluminescence Quantum Yield for Light-Emitting Diodes

Fang Yuan [1,†], Xiaoyun Liu [1,2,†], Songting Zhang [1], Peichao Zhu [1], Fawad Ali [1], Chenjing Zhao [1], Shuaiqi He [1], Qianhao Ma [1], Jingrui Li [3,*], Kunping Guo [4], Lu Li [1] and Zhaoxin Wu [1,5,*]

[1] Key Laboratory for Physical Electronics and Devices of the Ministry of Education & Shaanxi Key Lab of Information Photonic Technique, School of Electronic Science and Engineering, Xi'an Jiaotong University, Xi'an 710049, China; yuanf121@xjtu.edu.cn (F.Y.); fawad_ali@stu.xjtu.edu.cn (F.A.); shuaiqihe@stu.xjtu.edu.cn (S.H.)
[2] Branch of LONGi Green Energy Technology Co., Ltd. in XixianNew Area, No. 215 Jinggan Second Street, Yongle Town, Jinghe Xincheng, Xixian New Area, Xi'an 710018, China
[3] Electronic Materials Research Laboratory, Key Laboratory of the Ministry of Education, International Center for Dielectric Research and International Joint Laboratory for Micro/Nano Manufacturing and Measurement Technology, School of Electronic Science and Engineering, Xi'an Jiaotong University, Xi'an 710049, China
[4] School of Electronic Information and Artificial Intelligence, Shaanxi University of Science and Technology, Xi'an 710021, China
[5] Collaborative Innovation Center of Extreme Optics, Shanxi University, Taiyuan 030006, China
* Correspondence: jingrui.li@xjtu.edu.cn (J.L.); zhaoxinwu@mail.xjtu.edu.cn (Z.W.)
† These authors contributed equally to the work.

Experimental Section

Materials and chemicals: Copper iodide (CuI, 99.999%), dimethylsulfoxide (DMSO, ≥99.5%), TPBi and PEDOT: PSS (Baytron P VPAI 4083), dichloromethane, and anhydrous ethanol were purchased from Alfa Aesar. Tetraethylammonium iodide (TEAI, >98%) was purchased from TCI. Each precursor and solvent were used without further purification.

Single-crystal growth of $TEA_2Cu_2I_4$: Weigh 257.2 mg of tetraethylammonium iodide and 190.5 mg of cuprous iodide and place them in a 5 mL serum bottle. Add 2 mL of acetone and stir at 55°C for more than 4 hours. Then, filter the solution while it is still hot into a test tube, and after standing for 4 hours, colorless and transparent needle-like $TEA_2Cu_2I_4$ single crystals will be precipitated.

Characterizations: Single-crystal X-ray diffraction data were collected on a Bruker D8 Venture Photon II diffractometer with graphite-monochromated Ga Kα radiation (λ = 1.3414 Å) and Cu Kα radiation (λ = 1.5418 Å). An empirical absorption correction using SADABS was applied for all data. Powder X-ray diffraction (PXRD) data were obtained by a Bruker diffractometer. The absorption and PL spectra were obtained by a UV–vis spectrophotometer (HITACHI U-3010, Japan) and a fluorescence spectrometer (Fluoromax-4 spectrofluometer), respectively. The PLQY and temperature-dependent PL were tested using an Edinburgh FLS9 spectrometer from Xi'an Jiaotong University Analysis and Testing Center. Time-resolved PL was collected using an Optronis Optoscope streak camera system which had an ultimate temporal resolution of ~10 ps. Raman spectra were tested by a Laser Raman Spectrometer from Xi'an Jiaotong University Analysis and Testing Center. Thermogravimetric analyses (TGAs) were performed with a Hitachi STA7200 Thermal Analysis System at a heating rate of 10 °C/min in the range of 40–600 °C. The luminance–current–voltage (L–I–V) characteristics as well as the stability performance of the devices were measured using a computer-controlled sourcemeter (Keithley 2602) and

a calibrated silicon photodiode. All measurements were carried out under ambient conditions at room temperature.

***Fabrication of the $TEA_2Cu_2I_4$ thin film*:** The quartz / ITO substrates were cleaned successively with ITO cleaning solution, deionized water, acetone, and alcohol (1:1), and treated with UV–ozone for 10 min before use. The 0.3 M $TEA_2Cu_2I_4$ solution was prepared by dissolving TEAI and CuI (TEAI: CuI molar ratio of 1:1) in DMSO. The solution was stirred overnight at room temperature in a nitrogen-filled glove box. Subsequently, the $TEA_2Cu_2I_4$ thin films were formed by spin-coating the solutions at 2000 rpm for 70 s in the nitrogen-filled glove box.

***Blue LED Device Fabrication*:** The ITO-coated glass substrates were cleaned successively with ITO cleaning solution, deionized water, acetone, and alcohol (1:1), and treated with UV–ozone for 10 min before use. The PEDOT: PSS solution was spin-coated onto the ITO substrate at 1500 rpm for 30 s and then annealed at 130 °C for 20 min in ambient air. Subsequently, the $TEA_2Cu_2I_4$ films were prepared on the substrate by spin-coating the precursor solution (0.05 M). After that, the TPBi (55 nm), LiF (1 nm), and Al (100 nm) were deposited separately on the substrate by high-vacuum thermal evaporation.

***Devices Characterization*:** The luminance–current–voltage (*L-I-V*) characteristics of the devices were measured using a computer-controlled sourcemeter (Keithley 2602) and a calibrated silicon photodiode. All measurements were carried out at room temperature in a glove box with a 99.999% N_2 atmosphere to protect the devices from degradation by moisture and oxygen in atmospheric environment.

***DFT calculations*:** Both structural optimization and single-point calculations were performed using the tier-2 basis sets and a Γ-centered 6 × 4 × 4 k-point mesh. The PBEsol-optimized lattice constants were a = 8.57080 Å, b = 14.20100 Å, and c = 11.19830 Å, and the angles were $\alpha = \gamma = 90°$ and $\beta = 96.48°$. There was a small deviation of the DFT-optimized lattice from the one measured with single-crystal XRD in both size and shape, which is expected considering that DFT is actually performed at 0 K. The test calculations show that PBEsol gives the most appropriate lattice constants, outperforming other common exchange correlation functionals. Scalar relativistic effects were included via the zeroth-order regular approximation.

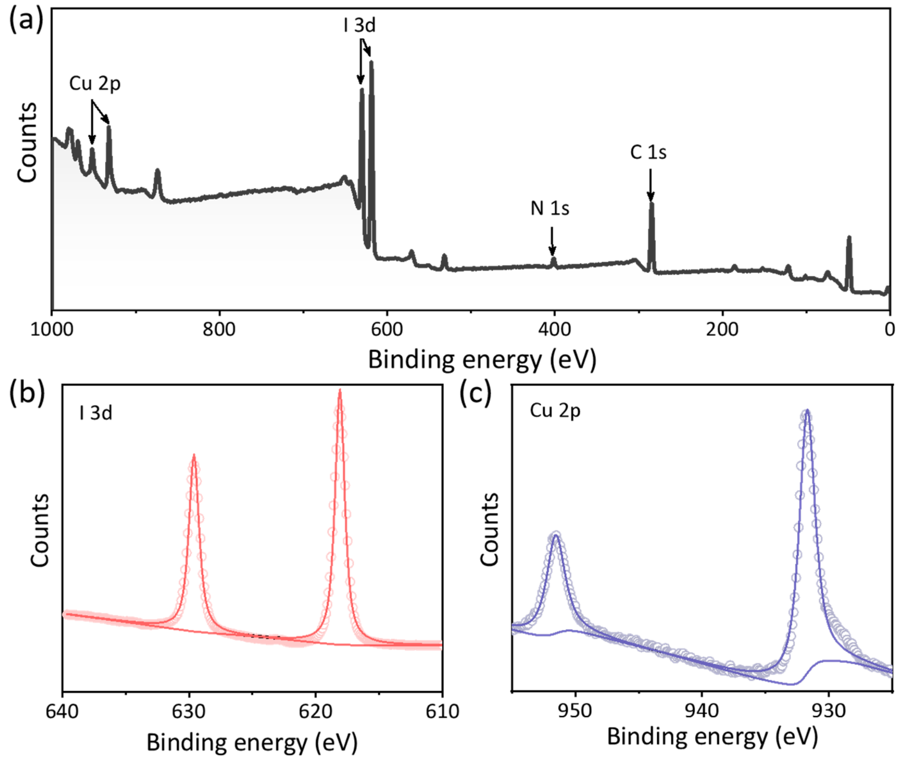

Figure S1. X-ray photoelectronic spectrum of $TEA_2Cu_2I_4$ film. (a) The total survey spectrum shows obvious characteristic peaks of C, N, I, and Cu elements, standardized by the peak of C. (b)-(c) show the I 3d and Cu 2p for $TEA_2Cu_2I_4$.

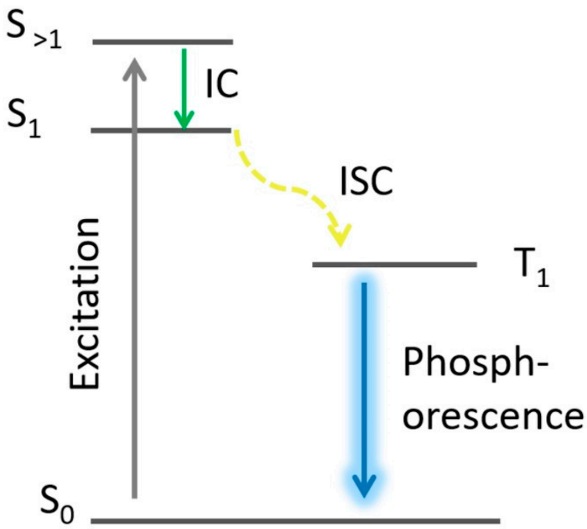

Figure S2. Proposed diagrams of phosphorescence emission process of $TEA_2Cu_2I_4$.

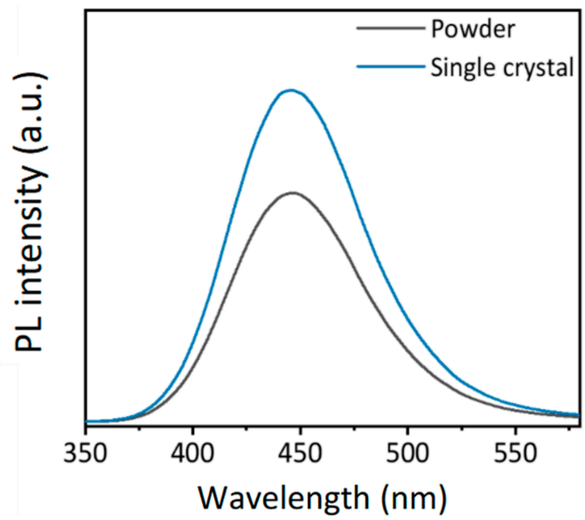

Figure S3. Comparison of PL spectra of $TEA_2Cu_2I_4$ single crystal and its powder after grinding.

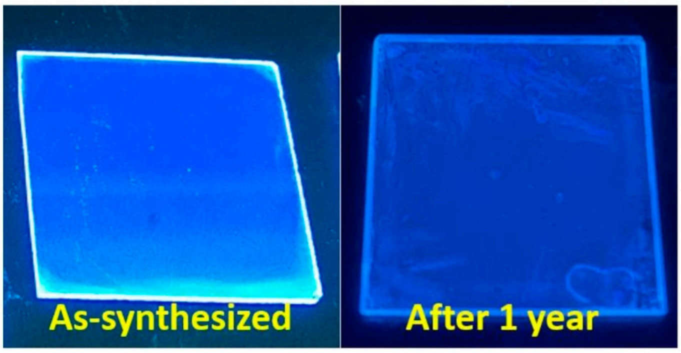

Figure S4. The contrast photos under ultraviolet light of the prepared $TEA_2Cu_2I_4$ polycrystalline thin film stored in air for one year.

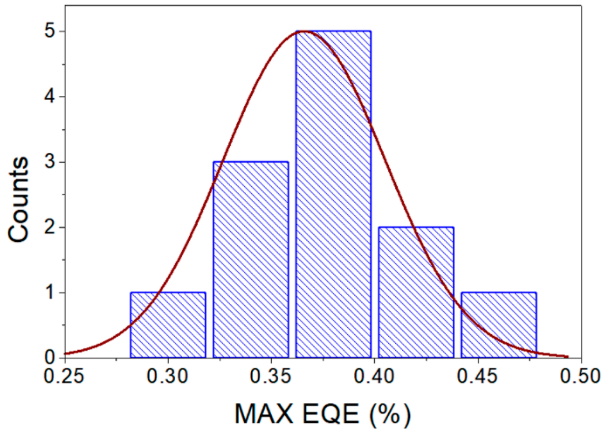

Figure S5. Histogram of maximum EQE for 12 devices from four batches.

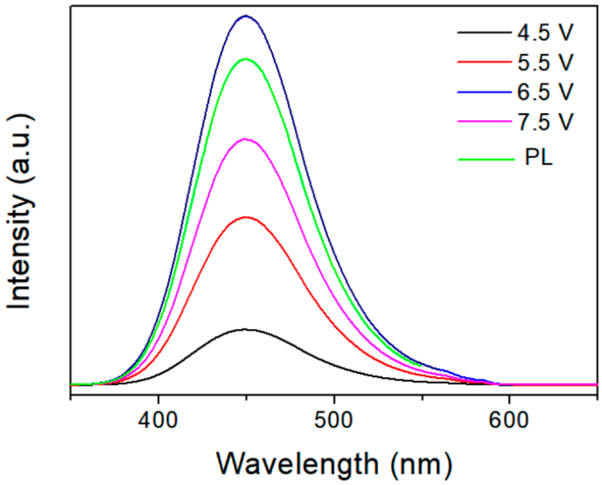

Figure S6. PL spectrum of $TEA_2Cu_2I_4$ film and EL spectra of $TEA_2Cu_2I_4$-dased LED device operating under different voltages.

Table S1. Detailed crystal parameters of the prepared $TEA_2Cu_2I_4$ SCs derived from single-crystal X-ray diffraction (SCXRD) data collected at 150 K.

	**Crystal data structure refinement**
Empirical formula	[N(C2H5)4]2Cu2I4
Empirical formula	447.59
Temperature	150(2) K
Wavelength	0.71073 A
Crystal system, space group	Monoclinic P 21/C
Unit cell dimensions	a = 8.5708(8) A alpha = 90 degb = 14.2010(13) A beta = 96.477(4) degc = 11.1983(11) A gamma = 90 deg
Volume	1354.3(2) A^3
Z, Calculated density	4, 2.195 Mg/m^3
Absorption coefficient	6.133 mm^{-1}
F(000)	840
Crystal size	0.98x 0.21 x 0.2 mm
Theta range for data collection	2.33 to 25.72 deg.
Limiting indices	-10<=h<=10, -17<=k<=17, -13<=l<=13
Reflections collected / unique	26626 / 2546 [R(int) = 0.0704]
Completeness to theta = 55.57	98.6 %
Data / restraints / parameters	2546 / 0 / 110
Goodness-of-fit on F^2	1.146
Final R indices [I>2sigma(I)]	R1 = 0.0498, wR2 = 0.1341
R indices (all data)	R1 = 0.0510, wR2 = 0.1349
Extinction coefficient	0.0069(7)
Largest diff. peak and hole	1.398 and -1.704 $e.A^{-3}$

Table S2. The atomic coordinates (× 10^4) and equivalent isotropic displacement parameters (A^2 × 10^3) for $TEA_2Cu_2I_4$.

	**X**	**y**	**z**	**U (eq)**
**Cu(1)**	4148(2)	360(1)	9049(1)	41(1)
**I(1)**	2701(1)	1010(1)	7156(1)	25(1)
**I(2)**	6391(1)	1309(1)	10215(1)	28(1)
**C(1)**	6442(10)	-1035(6)	7223(8)	26(2)
**C(2)**	5395(12)	-1204(7)	6071(10)	37(2)
**C(3)**	8305(11)	43(6)	6296(8)	26(2)
**C(4)**	7598(13)	957(7)	6714(10)	35(2)
**C(5)**	9004(11)	-620(6)	8322(8)	27(2)

**C(6)**	8992(12)	-1418(7)	9214(9)	34(2)
**C(7)**	8880(11)	-1645(6)	6499(8)	26(2)
**C(8)**	10649(11)	-1628(7)	6493(9)	32(2)
**N(1)**	8151(8)	-814(5)	7093(6)	20(1)

Table S3. Statistics of bond length and bond angle of $TEA_2Cu_2I_4$ single crystals.

**Bond**	**Lengths(Å)/angles(°)**	**Bond**	**Lengths(Å)/angles(°)**
Cu(1)-I(1)	2.5074(14)	I(1)-Cu(1)-I(2)	120.37(6)
Cu(1)-I(2)#1	2.5691(15)	I(2)#1-Cu(1)-I(2)	118.06(5)
Cu(1)-I(2)	2.5790(15)	I(1)-Cu(1)-Cu(1)#1	175.66(9)
Cu(1)-Cu(1)#1	2.649(3)	I(2)#1-Cu(1)-Cu(1)#1	59.21(5)
I(2)-Cu(1)#1	2.5691(15)	I(2)-Cu(1)-Cu(1)#1	58.84(5)
C(1)-N(1)	1.521(11)	Cu(1)#1-I(2)-Cu(1)	61.94(5)
C(1)-C(2)	1.505(13)	N(1)-C(1)-C(2)	116.1(8)
C(3)-N(1)	1.524(10)	N(1)-C(3)-C(4)	115.8(7)
C(3)-C(4)	1.527(12)	N(1)-C(5)-C(6)	115.3(7)
C(5)-N(1)	1.509(11)	N(1)-C(7)-C(8)	116.7(7)
C(5)-C(6)	1.511(13)	C(3)-N(1)-C(5)	108.9(6)
C(7)-N(1)	1.523(10)	C(3)-N(1)-C(1)	111.7(7)
C(7)-C(8)	1.517(13)	C(5)-N(1)-C(1)	108.9(6)
I(1)-Cu(1)-I(2)#1	121.39(6)	C(3)-N(1)-C(7)	107.2(6)
C(5)-N(1)-C(7)	111.0(7)	C(1)-N(1)-C(7)	109.2(6)
